# Factors Influencing Abortion Among Brothel-Based Female Sex Workers in Rural West Bengal, India

**DOI:** 10.7759/cureus.87333

**Published:** 2025-07-05

**Authors:** Aniruddha Biswas, Sanjoy K Sadhukhan, Jayita Pal, Rama R Pati, Protim Ray, Rajdeep Shaw, Nilima Saren

**Affiliations:** 1 Epidemiology, All India Institute of Hygiene and Public Health, Kolkata, IND; 2 Maternal and Child Health, All India Institute of Hygiene and Public Health, Kolkata, IND; 3 Medicine, Durbar Mahila Samanwaya Committee (DMSC), Kolkata, IND; 4 Preventive and Social Medicine, All India Institute of Hygiene and Public Health, Kolkata, IND

**Keywords:** condom use, female sex workers, reproductive health, rural india, unintended pregnancy

## Abstract

Introduction: Female sex workers (FSWs) face significant reproductive health challenges, including inconsistent condom use and high rates of unintended pregnancies and abortions. Understanding factors influencing these outcomes, particularly in rural brothel settings, is essential for targeted interventions.

Methods: A cross-sectional study was conducted among brothel-based FSWs in a rural region. Data on sociodemographic characteristics, sexual and contraceptive behaviors, and reproductive health outcomes were collected through structured interviews. Multivariate logistic regression was used to identify determinants of irregular condom use and abortion.

Results: The study included 210 FSWs, aged 19-49 years (mean 29.3 ± 5.6). All reported consistent condom use with clients; however, only 25 (18.8%) used condoms regularly with cohabiting partners. Among those with irregular condom use in these relationships, 74 (64.4%) cited trust as the main reason. Condom breakage was reported by 177 participants (84.3%), with most responding by washing genitalia only, and only 6(3.5%) using emergency contraception. Multivariate analysis identified higher daily income (>₹2000) (AOR=2.22, 95% CI:1.12-4.40), history of STDs/reproductive tract infections (RTIs) (AOR=1.93, 95% CI:1.08-3.45), and condom breakage (AOR=2.49, 95% CI:1.10-5.64) as significant predictors of irregular condom use. Abortion was significantly associated with cohabitation (AOR=7.14, 95% CI:2.11-24.12), sex work duration ≥3.5 years (AOR=2.09, 95% CI:1.03-4.26), and sex without alcohol use (AOR=3.03, 95% CI:1.25-7.31).

Conclusion: Irregular condom use and abortion among rural FSWs are influenced by economic, behavioral, and relational factors. Interventions focusing on improving contraceptive access, counselling on dual protection, and addressing the dynamics of intimate partnerships are critical to enhancing reproductive health outcomes in this vulnerable group.

## Introduction

Access to safe and legal abortion remains a critical component of reproductive rights; however, despite the Medical Termination of Pregnancy (MTP) Act legalizing abortion in India since 1971, access remains inequitable for marginalized groups such as female sex workers (FSWs) [1]. FSWs frequently encounter reproductive health challenges including unwanted pregnancies, restricted contraceptive options, and unsafe abortion procedures-often exacerbated by socio-cultural stigma, economic vulnerabilities, and healthcare discrimination [2-4]. Studies show that globally, FSWs experience higher rates of unintended pregnancies and abortion than the general female population due to inconsistent condom use, contraceptive failure, lack of negotiation power, and client refusal [5,6]. Unsafe abortions continue to be a significant contributor to maternal morbidity and mortality, particularly when carried out in unregulated settings or by unqualified providers [7].

Previous research in India has primarily focused on urban sex workers, particularly in metropolitan areas [8,9]. In contrast, data on abortion-related factors among FSWs in rural areas remain limited. Rural FSWs, often outside formal brothel systems, lack adequate access to healthcare infrastructure and reproductive services, with minimal support from NGOs and community organizations [10,11]. In West Bengal, rural districts such as North 24 Parganas, Murshidabad, and Nadia are known for hidden FSW populations with limited mobility and access to reproductive health resources, despite governmental and non-governmental efforts through initiatives like the National Health Mission [12].

To date, no community-based studies have comprehensively examined the determinants of induced abortion among FSWs in rural West Bengal. This research aims to fill that gap by identifying sociodemographic, occupational, and reproductive health-related variables associated with abortion in this marginalized group. The findings are anticipated to support targeted, evidence-based reproductive health interventions.

## Materials and methods

Study design and area

A community-based observational approach was adopted using a cross-sectional study design to determine the prevalence of induced abortion specifically after joining the profession and associated factors among FSWs operating within brothels. The research was carried out in Matia Bazar, located in the Basirhat-II block along Taki Road, roughly 60 kilometers from Kolkata, West Bengal, a red-light area. Red-light areas are designated urban zones or neighborhoods where sex work is practiced openly [13]. This red-light area includes nearly 100 functioning brothels. The Durbar Mahila Samanwaya Committee (DMSC) facilitates local support services through a dedicated drop-in center and clinic.

Study population and eligibility criteria

The study targeted brothel-based FSWs who were officially enrolled with the DMSC and had resided in the study location for at least one year before the commencement of the study. Women who were either critically unwell or declined to participate were not considered for inclusion.

Sample size and sampling procedure

Drawing upon prior data that estimated lifetime pooled prevalence of abortion among FSWs, as per a systematic review and meta-analysis conducted in 2023, at around 37.7%, the required sample size was calculated as 185 participants to achieve a 95% confidence interval and a 7% precision [5]. To accommodate a possible 10% non-response rate, the sample size was adjusted upward to 204; rounding it off, the researchers finalized the sample size to 210. A list of all eligible participants was compiled using DMSC registration records. From this list, 210 individuals were selected using a simple random sampling method without replacement.

Study instruments and data collection techniques

An interview schedule was initially crafted in English and then translated into Bengali by a language expert, ensuring linguistic and cultural relevance. To validate the translation, two independent researchers, unaware of the original content, retranslated it into English. The instrument’s face and content validity were verified by experienced researchers and public health professionals. Reliability was confirmed using the test-retest approach. Pilot testing was performed, and the tool was refined accordingly before full-scale data collection. Data collection was conducted through direct, in-person interviews after obtaining voluntary written consent from each participant.

Field procedure

The principal investigator, accompanied by a peer educator affiliated with the DMSC (serving solely as a facilitator), visited the homes of selected FSWs. After briefing participants on the study objectives, informed consent was obtained. Interviews and relevant clinical examinations were conducted in the presence of a female attendant to ensure participant comfort and safety. In cases where a selected participant was unavailable, up to two follow-up visits were made. If a participant declined or was found ineligible, the nearest eligible FSW was approached as a replacement.

To address the sensitivity of abortion-related topics and reduce interviewer and social desirability bias, several safeguards were implemented. The presence of a trusted peer educator helped foster a secure environment and encouraged open communication. Interviewers were specially trained in ethical data collection practices, confidentiality assurance, and non-judgmental interviewing techniques. Data collection was carried out in private and familiar settings, such as NGO drop-in centers or discreet community spaces. Participants were clearly assured of anonymity and informed that their responses would remain confidential and have no personal repercussions. The interview schedule was designed with neutral, non-leading questions, and indirect questioning techniques were used for particularly sensitive topics. Efforts were made to build rapport early in the interaction to facilitate candid disclosure.

Information was collected on participants' socio-demographic, behavioral, and occupational characteristics, contraceptive use, abortion history, and self-reported reproductive tract infection (RTI)/ sexually transmitted infection (STI) symptoms within the past year. Throughout the study, strict adherence to ethical and confidentiality protocols was maintained.

Ethical clearance

It was obtained from both from Institutional Ethics Committee [IEC/2023(1)/14] and Durbar Ethics Review Board [DERB-02/2023].

Data analysis

Collected data were entered into Microsoft Excel (2021; Microsoft Corporation, Redmond, USA) and subsequently analyzed using Jamovi software (version 2.5.3). Descriptive statistics including mean values, standard deviations, frequencies, and proportions were computed and organized in tables. For inferential statistics, chi-square or Fisher’s exact tests were applied for categorical variables, while t-tests or ANOVA were employed for continuous variables. A p-value less than 0.05 was considered statistically significant, and all tests were two-tailed. Bivariate analyses were initially performed to explore associations between various factors and STD presence. Variables that showed significant relationships were included in a multivariable logistic regression model to identify independent predictors. Multicollinearity was assessed using the variance inflation factor (VIF) values prior to model fitting. All variables retained in the final model had VIF values below the commonly accepted threshold of 2, indicating low multicollinearity.

## Results

Sociodemographic profile

The age of participants ranged from 19 to 49 years, with a mean age of 29.3 years (±5.6) and a median of 29 years. A majority, 127(60.5%), were in the 21-30-year age group. Regarding marital status, 91(43.3%) were divorced or separated, and 84(40%) were currently married.

In terms of cohabitation, 77(36.7%) reported living without a partner, 75(35.7%) resided with their husbands, 49(23.3%) lived with a babu (non-marital partner), and 9(4.3%) lived with both a husband and a babu. Educational levels varied: 70(33.3%) had completed middle school, 51(24.3%) had primary education, 34(16.2%) had never attended formal school, and only 8(3.8%) had studied beyond higher secondary level.

In addition to sex work, 29(13.8%) of participants engaged in supplementary income-generating activities such as domestic labor, tailoring, or small-scale business.

Nearly half of the participants, 103(49%), reported having two children. The average number of children per participant was 1.52 (±0.8), and the average number of dependents was 3.96 (±1.3), with most supporting three or more individuals.

Daily income among participants ranged from ₹400 to ₹9000, with a mean income of ₹2104 (±1176) and a median income of ₹2000. About 128(61%) reported earning ₹2000 or less per day.

Substance use was reported by 114(54.2%) of participants. Smoking was reported by 62(29.5%), with 48(22.8%) having smoked for five years or less. Use of smokeless tobacco was noted in 50(23.8%) of participants, of whom 30(14.3%) had been using it for more than five years. Alcohol consumption was reported by 58(27.6%), with 44(20.9%) drinking for five years or less (Table 1).

**Table 1:** Distribution of FSWs according to sociodemographic and behavioural characteristics (n=210) FSWs: Female sex workers

Category	Sub-category	No. (%)
Age group (years)	Up to 20	8 (3.8%)
	21–25	50 (23.8%)
	26–30	77 (36.7%)
	31–35	49 (23.3%)
	36–40	20 (9.5%)
	Above 40	6 (2.9%)
Marital status	Never married	10 (4.8%)
	Currently married	84 (40.0%)
	Widowed	25 (11.9%)
	Separated/Divorced	91 (43.3%)
Cohabiting status	None	77 (36.7%)
	Husband	75 (35.7%)
	Babu	49 (23.3%)
	Both	9 (4.3%)
Educational status	No formal education	34 (16.2%)
	Below primary	21 (10.0%)
	Primary	51 (24.3%)
	Middle school	70 (33.3%)
	Secondary	26 (12.4%)
	Higher secondary and above	8 (3.8%)
Additional occupation	Maidservant	10 (4.8%)
	Petty business	6 (2.9%)
	Non-agricultural labor	4 (1.9%)
	Tailor	4 (1.9%)
	Bar dancer	3 (1.4%)
	Peer educator	2 (1.0%)
No. of living children	0	24 (11.4%)
	1	68 (32.4%)
	2	103 (49.0%)
	≥3	15 (7.2%)
No. of family members	0	3 (1.4%)
	1	4 (1.9%)
	2	17 (8.1%)
	3	40 (19.0%)
	4	79 (37.6%)
	5	49 (23.4%)
	≥6	18 (8.6%)
Daily income (in ₹)	Up to 2000	128 (61.0%)
	2001–4000	76 (36.1%)
	Above 4000	6 (2.9%)
Smoker	Total Smokers	62 (29.5%)
	Up to 5 Years	48 (22.8%)
	Above 5 Years	14 (6.7%)
Smokeless Tobacco Users	Total users	50 (23.8%)
	Up to 5 years	30 (14.3%)
	Above 5 years	20 (9.5%)
Alcohol Users	Total users	58 (27.6%)
	Up to 5 years	44 (20.9%)
	Above 5 years	14 (6.7%)

Occupational characteristics

The most frequently reported age of entry into sex work was between 21 and 25 years, accounting for 95(45.2%) of participants. The overall age range at initiation into the profession spanned from 15 to 39 years, with a mean age of 24.1 years (±4.7). A majority, 149(71.0%), had been engaged in sex work for five years or less. The mean duration in the profession was 5.1 years (±4.6), while the median duration was 3.5 years, with an interquartile range (IQR) of 4.8 years.

In terms of work intensity, participants reported serving an average of 9.6 clients per day and being professionally active for an average of 19.1 days per month (±3.4). The number of clients served monthly varied significantly, ranging from 30 to 552, with a median of 155 clients (IQR: 152).

High-risk practices were notable among the participants. Body modifications, including tattoos and piercings, were reported by 87(41.4%) of respondents. Additionally, 55(26.2%) reported engaging in sexual activity while under the influence of alcohol. Regarding non-vaginal sexual behaviors, 38(18.1%) of participants acknowledged participating in alternative practices; of these, 32(15.2%) engaged exclusively in oral sex, while 6(2.9%) reported both oral and anal sexual activities (Table 2).

**Table 2:** Distribution of FSWs according to occupational and high-risk behavioral characteristics (n=210) FSWs: Female sex workers

Category	Sub-category	No. (%)
Occupational profile		
Age of joining the profession	Below 20	54 (25.7%)
	21–25	95 (45.2%)
	26–30	34 (16.2%)
	31–35	22 (10.5%)
	36–39	5 (2.4%)
Duration in profession (years)	1–5	149 (71.0%)
	6–10	38 (18.1%)
	>10	23 (11.0%)
Clients per month	≤100	59 (28.1%)
	101–200	78 (37.2%)
	201–300	45 (21.4%)
	301–400	15 (7.1%)
	>400	13 (6.2%)
High-risk behavioral practices	Tattooing/body piercing	87 (41.4%)
	Sex under influence of alcohol	55 (26.2%)
	Sexual intercourse other than vaginal	38 (18.1%)
	Only oral	32 (15.2%)
	Oral and anal	6 (2.9%)

Contraceptive practices and reproductive health outcomes. While all FSWs reported consistent condom use with clients, only 25(18.8%) did so with their live-in partners. Among the 115(86.5%) who admitted to inconsistent condom use in such relationships, the primary reason cited by 74(64.4%) was a sense of trust in their partners. Within the past six months, 177(84.3%) of participants had experienced at least one incident of condom breakage. In response to these incidents, the majority, 139(78.5%), resorted to washing their genital area, whereas only 6(3.5%) used emergency contraception.

Awareness of female condoms was notably low, with just 35(16.7%) of respondents reporting knowledge of them. Only one participant had attempted to use a female condom but discontinued due to a partner’s unwillingness to cooperate.

Use of alternative contraceptive methods was variable. Oral contraceptive pills were used by 62(29.5%) of participants, 41(19.5%) had undergone sterilization, 20(9.5%) used intrauterine contraceptive devices (IUCDs), 17(8.1%) reported using emergency contraception, and 2(1.0%) used injectable contraceptives (Table 3).

**Table 3:** Distribution of FSWs according to contraceptive practices (n=210) FSWs: Female sex workers

Category	Sub-category / Description	No. (%)
Condom use (self-reported)		
Last intercourse with a paying partner	Used condom	210 (100.0%)
Last intercourse with cohabiting partner (n=133)	Used condom	25 (18.8%)
Condom use with a cohabiting partner in the last two weeks (n=133)		
Frequency of use	Regular use	18 (13.5%)
	Irregular use	115 (86.5%)
Reasons for irregular condom use (n=115)		
Main reason	Trust in partner	74 (64.4%)
	Partner’s refusal to use condoms	16 (13.9%)
	Use of other contraceptive methods	11 (9.6%)
	Permanent sterilization	9 (7.8%)
	Participant unwilling to use condoms	5 (4.3%)
Experience of condom breakage	Ever experienced breakage (includes intentional tearing)	177 (84.3%)
Post-condom breakage actions taken (n=177)		
Response	Took no action	21 (11.8%)
	Washed genitals only	139 (78.5%)
	Took emergency contraceptive (EC) pill only	11 (6.2%)
	Washed genitals and took EC pill	6 (3.5%)
Female condom awareness and use	Heard about a female condom	35 (16.7%)
	Ever used a female condom	1 (0.5%)
Use of other contraceptives	Oral contraceptive pill (OCP)	62 (29.5%)
	Permanent sterilization	41 (19.5%)
	Intrauterine contraceptive device (IUCD)	20 (9.5%)
	Emergency contraceptive (EC)	17 (8.1%)
	Injectable contraceptive	2 (1.0%)

The study revealed that more than one-quarter (60; 28.6%) of participants reported having undergone an abortion after entering the sex trade, with induced abortions accounting for the majority, 42(20.0%). Furthermore, 94(44.8%) of respondents reported experiencing at least one STI or RTI in the past year.

Multivariate logistic regression analysis identified several significant predictors of inconsistent condom use among FSWs. Participants with a higher daily income (>₹2000) had more than twice the odds of inconsistent condom use compared to those earning less (AOR = 2.22; 95% CI: 1.12-4.40). A self-reported history of sexually transmitted or reproductive tract infections (STDs/RTIs) was also significantly associated with inconsistent condom use (AOR = 1.93; 95% CI: 1.08-3.45). Furthermore, participants who reported experiences of condom breakage had significantly higher odds of inconsistent use (AOR = 2.49; 95% CI: 1.10-5.64).

The overall model demonstrated a Nagelkerke R² value of 0.13, indicating that 13% of the variance in inconsistent condom use was explained by the included variables. The model also demonstrated good fit, as indicated by the Hosmer-Lemeshow goodness-of-fit test (p = 0.797) (Table 4).

**Table 4:** Factors associated with condom use among FSWs (n=210) FSWs: Female sex workers; STI: sexually transmitted infection; RTI: reproductive tract infection

Characteristic	Category (n)	Irregular Use No. (%)	OR (95% CI)	AOR (95% CI)
Age (in years)	Below 29 (100)	61 (61.0%)	1.62 (0.94–2.81)	–
	29 and above (110)	54 (49.1%)	1 (Reference)	1 (Reference)
Educational status	Up to primary (106)	52 (49.1%)	0.63 (0.362–1.08)	–
	More than primary (104)	63 (60.6%)	1 (Reference)	1 (Reference)
Income per day (INR)	Above 2000 (82)	56 (68.3%)	2.52 (1.41–4.50)	2.22 (1.12–4.40)
	Up to 2000 (128)	59 (46.1%)	1 (Reference)	1 (Reference)
Any substance use	Yes (114)	63 (55.3%)	1.05 (0.61–1.80)	–
	No (96)	52 (54.2%)	1 (Reference)	1 (Reference)
Duration in profession	≥3.5 years (105)	64 (61.0%)	1.65 (0.96–2.86)	–
	<3.5 years (105)	51 (48.6%)	1 (Reference)	1 (Reference)
Clients per month	≥155 (105)	66 (62.9%)	1.93 (1.11–3.36)	1.13 (0.58–2.22)
	<155 (105)	49 (46.7%)	1 (Reference)	1 (Reference)
Sex under the influence of alcohol	Yes (55)	33 (60.0%)	1.34 (0.72–2.49)	–
	No (155)	82 (52.9%)	1 (Reference)	1 (Reference)
Tattooing/body piercing	Present (87)	53 (60.9%)	1.53 (0.88–2.68)	–
	Absent (123)	62 (50.4%)	1 (Reference)	1 (Reference)
Non-vaginal sexual intercourse	Yes (38)	26 (68.4%)	2.02 (0.96–4.26)	–
	No (172)	89 (51.7%)	1 (Reference)	1 (Reference)
History of STI/RTI	Yes (94)	61 (64.9%)	2.12 (1.21–3.71)	1.93 (1.08–3.45)
	No (116)	54 (46.6%)	1 (Reference)	1 (Reference)
Condom breakage history	Yes (177)	103 (58.2%)	2.44 (1.13–5.26)	2.49 (1.10–5.64)
	No (33)	12 (36.4%)	1 (Reference)	1 (Reference)

Multivariate logistic regression analysis identified several significant predictors of having undergone an induced abortion among female sex workers. Participants who reported cohabiting with a partner had markedly higher odds of abortion compared to those without a cohabiting partner (AOR = 7.14; 95% CI: 2.11-24.12). Being engaged in sex work for 3.5 years or more was also significantly associated with increased odds of abortion (AOR = 2.09; 95% CI: 1.03-4.26). Interestingly, participants who engaged in sexual activity without the influence of alcohol showed significantly greater likelihood of having had an abortion (AOR = 3.03; 95% CI: 1.25-7.31).

The model explained 16% of the variance (Nagelkerke R² = 0.16) and had a good fit (Hosmer-Lemeshow p = 0.736) (Table 5).

**Table 5:** Factors associated with abortion after joining this profession among FSWs (n=210) FSWs: Female sex workers

Characteristic	Category (n)	Abortion No. (%)	OR (95% CI)	AOR (95% CI)
Age (in years)	Below 29 (100)	39 (35.5%)	2.07 (1.11–3.84)	1.65 (0.78–3.47)
	29 and above (110)	21 (21.0%)	1 (Reference)	1 (Reference)
Cohabiting partner	Present (133)	46 (34.6%)	2.38 (1.21–4.70)	7.14 (2.11–24.12)
	Absent (77)	14 (18.2%)	1 (Reference)	1 (Reference)
Educational status	Up to primary (106)	34 (32.1%)	1.42 (0.76–2.59)	–
	More than primary (104)	26 (25.0%)	1 (Reference)	1 (Reference)
Income per day (INR)	Up to 2000 (128)	43 (33.6%)	1.93 (1.01–3.70)	1.26 (0.58–2.73)
	Above 2000 (82)	17 (20.3%)	1 (Reference)	1 (Reference)
Any substance use	Yes (114)	36 (31.6%)	1.39 (0.75–2.54)	–
	No (96)	24 (25.0%)	1 (Reference)	1 (Reference)
Duration in profession (years)	≥3.5 years (105)	39 (37.1%)	2.36 (1.27–4.40)	2.09 (1.03–4.26)
	<3.5 years (105)	21 (20.0%)	1 (Reference)	1 (Reference)
Clients per month	≥155 (105)	30 (28.6%)	1.00 (0.60–1.82)	–
	<155 (105)	30 (28.6%)	1 (Reference)	1 (Reference)
Sex during alcohol use	Yes (55)	10 (18.2%)	2.14 (1.00–4.59)	3.03 (1.25–7.31)
	No (155)	50 (32.3%)	1 (Reference)	1 (Reference)
Tattooing/body piercing	Present (87)	29 (33.3%)	1.48 (0.81–2.71)	–
	Absent (123)	31 (25.2%)	1 (Reference)	1 (Reference)
Non-vaginal sexual intercourse	Yes (38)	7 (18.4%)	1.97 (0.82–4.76)	–
	No (172)	53 (30.8%)	1 (Reference)	1 (Reference)
Condom use	Irregular (115)	37 (32.2%)	1.49 (0.81–2.74)	–
	Regular (95)	23 (24.2%)	1 (Reference)	1 (Reference)
Condom breakage	Yes (177)	56 (31.6%)	3.36 (1.13–10.0)	2.81 (0.90–8.78)
	No (33)	4 (12.1%)	1 (Reference)	1 (Reference)

## Discussion

This study provides valuable insights into the reproductive and sexual health behaviors of brothel-based FSWs in Matia Bazar, Basirhat, highlighting key determinants of irregular condom use and abortion. The findings reveal significant behavioral patterns and risk factors underscoring the contextual nuances of this population.

Despite universal condom use with clients, only 25 (18.8%) of respondents reported consistent condom use with live-in partners. Trust was the most frequently cited reason for non-use, mirroring findings from multiple Indian studies. Like the studies conducted in Kolkata and Mumbai, emotional bonding and trust often override risk perceptions in non-commercial relationships [14,15]. In Kolkata’s Sonagachi red-light area, similar dynamics were observed, where FSWs perceived condom use with regular partners as a sign of distrust, impacting negotiation [14].

In the present study, higher daily income, a history of STDs/RTIs, and prior condom breakage were independently associated with irregular condom use. Previous research found a correlation between higher income and inconsistent condom use-possibly due to better-paying clients influencing FSWs to compromise on safety. This study observed that FSWs with higher income may prioritize client preferences or perceive reduced personal risk [16,17]. Supporting evidence from Kolkata indicated that FSWs with financial independence were less strict in condom negotiations, particularly with regular clients [18]. Likewise, research in Karnataka suggested that economic empowerment without risk perception education might lead to complacency in condom use [19].

A history of STIs or RTIs has also been associated with inconsistent condom use and was found to predict future irregular use in studies from Andhra Pradesh and Maharashtra [20,21]. These studies suggested that FSWs with prior STDs were more prone to high-risk behaviors due to structural vulnerabilities, low empowerment, and inadequate risk perception.

Condom breakage, another significant determinant, undermines confidence in condom efficacy. A study in Bangalore found that breakages were often due to incorrect usage and insufficient demonstrations, leading to reduced trust and future use [22].

The study findings underscore the need for reproductive health services that address the specific challenges faced by FSWs in cohabiting relationships and with long-term occupational exposure. Although Durbar-affiliated NGO clinics operate in the area with a focus on STI and HIV services, they lack the capacity to manage abortion care and general health issues such as acute or chronic illnesses. Consequently, many FSWs rely on local private providers or distant government facilities, where they frequently encounter stigma, discrimination, and breaches of confidentiality. Fear of exposure often compels them to conceal their occupation or use false identities, leading to delayed or inadequate care. Strengthening NGO clinic capacity, integrating non-stigmatizing abortion services, and sensitizing local government providers are essential. Mobile health units, peer-led referral support, and formal linkages between NGO and public health systems can enhance accessibility and reduce unsafe abortions in this underserved population.

In the current study, abortion was significantly associated with cohabiting partnerships, longer duration in sex work (≥3.5 years), and engagement in sex without alcohol use. As noted in prior multi-site Indian studies, cohabitation with intimate or non-paying partners has been linked to lower condom use and higher rates of unintended pregnancies and abortions [20]. Similarly, Fehrenbacher et al. reported that cohabiting FSWs often face greater difficulty negotiating contraception with non-paying partners [16].

Longer involvement in sex work (≥3.5 years) may indicate increased exposure to occupational risks and decreased access to comprehensive reproductive health services. Fehrenbacher et al. also found that FSWs working for longer durations had higher rates of pregnancy and abortion, likely due to repeated exposure and shifting dynamics in condom negotiation [16]. Additionally, older FSWs may experience declining client demand, leading to riskier engagements and diminished control over contraceptive use [4].

Interestingly, this study found a positive association between abortion and engaging in sex without alcohol, a result that appears counterintuitive. However, FSWs who abstain from alcohol during sex may be more likely to be in stable, non-commercial relationships with lower condom use and hence higher pregnancy risk. Supporting evidence from southern India found significantly lower condom use with non-paying partners compared to clients due to trust and emotional intimacy [22].

The Nagelkerke R² values for the regression models were modest (13-16%). This is not uncommon in behavioral and reproductive health research, especially when dealing with complex, multi-level factors among marginalized populations like FSWs. Many unmeasured psychosocial, economic, and contextual variables, such as stigma, coercion, access barriers, or prior trauma, may influence abortion decisions but were beyond the scope of this study. Despite the low R², the significant associations identified still offer valuable insights and generate hypotheses for further research.

These findings call for tailored reproductive health services addressing the specific needs of FSWs in cohabiting relationships and those with prolonged occupational exposure. Dual protection counselling, better access to modern contraception, and non-stigmatizing abortion services are crucial to mitigate unintended pregnancies and unsafe abortions in this vulnerable population.

This study has limitations. Its cross-sectional design restricts causal inference between associated factors and outcomes like abortion or irregular condom use. Self-reported data may suffer from recall or social desirability bias. The geographic limitation to brothel-based FSWs in one rural area reduces generalizability. A small sample size may have limited the statistical power to detect some associations. Moreover, critical structural factors such as healthcare access and legal context were not explored, though they likely influence reproductive outcomes

## Conclusions

This study highlights important factors associated with irregular condom use and abortion among brothel-based FSWs in a rural setting. Higher income, history of STDs/RTIs, and condom breakage increase the risk of inconsistent condom use, while cohabitation, longer duration in sex work, and sex without alcohol are linked to higher abortion rates. These findings emphasize the need for tailored reproductive health interventions that address the unique challenges faced by FSWs, particularly in managing stable partnerships and occupational risks. Strengthening access to comprehensive contraceptive options, counselling on dual protection, and destigmatizing abortion services are essential to improve reproductive health outcomes in this vulnerable population.

## Appendices

Questionnaire

**Table 6:** Socio-economic information

1.1.	What is your age (in complete years)?	Age…………yrs.
1.2.	What education have you had? (record the highest level attained)	Illiterate (0) Below primary (including non-formal) (1) Primary (completed class IV) (2) Mid-schooling (completed class VIII) (3) Secondary (completed class X) (4) Higher secondary and above (completed class XII) (5)
1.3.	What is your current marital status?	Never married (1) Currently married (2) Widowed (3) Separated/Divorced (4)
1.4.	Do you have a cohabiting partner?	None (0) Husband (1) Lover (2) Both (3)
1.5.	Do you have children?	Yes (1) number…...……(specify) No (0)
1.6.	What is your average income/day? (in Rs.)	
1.7.	How much is the income of your family? ((in Rs.)	
1.8.	How many persons are dependent on it?	
1.9.	Apart from sex work, what other work do you do to earn?	None (0) Non-agricultural labour (1) Petty business (2) Agricultural labour (3) Maid servant (4) Others(specify) (9)……………

**Table 7:** Behavioral factors

2.1	Do you have the practice of tattooing or body piercing?		No (0) Yes (1)			
2.2	Addiction	Ever used	Currently Using		Age when you used for the first time	Age when you used for the last time
2.2.1	Smokeless Tobacco (snuff/chewed tobacco/Ghutka, etc.)	No (0) Yes (1) If yes, (specify the type and average amount /day) …… No. of days/wk…..	No (0) Yes (1) If yes, (specify the type and average amount /day) …… No. of days/wk…..			
2.2.2	Smoking (cigarettes, bids, hookah, etc.)	No (0) Yes (1) If yes, (specify the type and average amount /day) …… No. of days/wk…..	No (0) Yes (1) If yes, (specify the type and average amount /day) …… No. of days/wk…..			
2.2.3	Alcohol	No (0) Yes (1) If yes, (specify the type and average amount /day) …… No. of days/wk…..	No (0) Yes (1) If yes, (specify the type and average amount /day) …… No. of days/wk…..			
2.2.4	Others	No (0) Yes (1) If yes, (specify the type and average amount /day) …… No. of days/wk…..	No (0) Yes (1) If yes, (specify the type and average amount /day) …… No. of days/wk…..			
2.2.5	Do you have practice of injecting drugs for non-treatment purpose?			No (0) Yes (1)		
2.3	Do you continue sexual intercourse after drinking alcohol?			No (0) Yes (1)		
2.4	Do you practice intercourse other than vaginal route?			No (0) Yes (1)		If yes, Oral (1) Anal (2) Both (3)

**Table 8:** Occupational factors

3.1	How old were you when you first started working as a sex worker? (in completed years)	………yrs.
3.2	How long (in years) have you been working in this profession?	………yrs.
3.3	What is the average no. of clients entertained per day?	
3.4	What is the average no. of working days per month?	

**Table 9:** Practice regarding reproductive health

4.1	Are you currently using any contraceptives?	Yes (1) No (0) If yes, Condom (1) Male condom Female condom Both Birth control pill (2) Copper-T/IUD (3) Injection (4) Emergency contraceptive (5) Permanent sterilization (6) Other (9) …….………(specify)				
4.2	Did your partner use a condom during the last time you had intercourse?	Paying partner			Lover/ husband	
		Yes (1) No (0)			Yes (1) No (0)	
4.3.1	How often during the last two weeks has your paying partner used condoms with you?	Never (0) Sometime (2) Every time (3)				
4.3.2	If sometime/ never, then what was the main reason for not using a condom?	Client did not want to use (1) Condom not available (2) Trust the clients (3) Used other contraceptives (4) Other (9)………………(specify)				
4.4.1	How often during the last two weeks has your cohabiting partner used condoms with you?	Never (0) Sometime (2) Every time (3)				
4.4.2	If sometime/ never, then what was the main reason for not using a condom?	………………(specify)				
4.5.1	Do you have any experience with a condom breaking while it was being used?	No (0) Yes (1) Don’t know/ don’t remember (9)				
4.5.2	If yes, what did you do then?	Nothing (0) Washed genitalia only (1) Took medicine (2) Did blood test (3) Other (9)………..(specify)				
4.6.1	Have you heard of the female condom?	Yes (1) No (0)				
4.6.2	Have you ever used it?	Yes (1) No (0)	If no, why? Never seen (0) Could not afford (1) Felt inconvenient (2) Partner did not want to use (3) Other(9)….….(specify)			
4.7.1	What type of sanitary kit do you use?	New cloth piece (1) Old cloth piece (2) Sanitary napkin (3) Both {cloth & napkin} (4) Other (9)…………(specific)				
4.7.2	Cause of non-usage of sanitary napkins	Financial (1) Sometimes forgets to buy (2) Using a cloth piece as an old habit (3) Other (9)…………(specify)				
4.7.3	For old clothes	Do you use soap during washing?				Do you dry it in sun?
		No (0) Yes (1) Some time (2)				No (0) Yes (1) Some time (2)
4.8.1	Do you have to bathe during menstruation?	Never (0) Always (1) Irregular (2)				
4.8.2	Do you have privacy while taking a bath?	Yes (1) No (0)				
4.8.3	Is there adequate water supply? (average 4-5 buckets/person daily)	Yes (1) No (0)				
4.9	Do you continue sexual intercourse during menstruation?	Yes (1) No (0)		If no, reason Feel bad (1) Fear of bleeding / pain (2) Fear of pregnancy (3) Partner does not like (4) Other (9) ……….(specify)		

**Table 10:** Morbidity status and health-seeking behavior

5.1.1	Have you suffered from any of the following? Foul smelling vaginal discharge Genital ulcer /sore/itching Lower abdominal excessive pain during or in between periods Swelling in groin area Pain while passing urine Genital warts Fever along with any of these	Last two weeks		>Last two weeks to one year
		5.1.2	Where have you visited for your illness?	Government hospital (1) Private facility (2) Durbar clinic (3) AYUSH (4) No treatment sought (0) Other (9)………(specify)		
5.1.3	What is the present status of your illness?	Untreated (0) Under treatment (1) Cured (2)		
5.2.1	Did you suffer from any other disease apart from those mentioned above during…	Last two weeks		>Last two weeks to one year
		5.2.2	Where have you visited for your illness?	Government hospital (1) Private facility (2) Durbar clinic (3) AYUSH (4) No treatment sought (0) Other (9)…….…(specify)		
5.2.3	What is the present status of your illness?	Untreated (0) Under treatment (1) Cured (2)		
5.3	Have you tested for HIV within the last three months?	Yes (1) No (0)		
5.4	Have you tested any cervical cancer screening (VIA/Pap smear)?	Yes (1) No (0) Don’t know/ don’t remember (9)		
5.5	Are you vaccinated against HPV?	Yes (1) No (0) Don’t know/ don’t remember (9)		
5.6	Source of condom?	Durbar (1) Chemist shop (2) Both (3) Other (9)…….(specify)		
5.7	Where did you go for ligation? (for the respondents who underwent ligation)	(specify)……..		
5.7.1	Have you ever had any abortion?	No (0) Yes (1)	If yes, At………week. Spontaneous (1) Induced (2)	
5.7.2	Where did you go for abortion? (for the respondents who underwent abortion)	(specify)…….		

Clinical examination

  I.          *General Survey*

a.      Pulse rate: …………. /min

b.     BP: ……………. mmHg

c.      Pallor: present (1) / absent (0)

d.     Icterus: present (1) / absent (0)

e.      Lymph node:

§  Palpable lymph node: present (1) / absent (0)

§  Tenderness: present (1) / absent (0)

  II.        *Anthropometric Measurements*

a.      Height ………….cm

b.     Weight …………kg

  III.     *Systemic Examination*

a.      Nervous system:

b.     Respiratory system:

c.      Cardiovascular system:

d.     G.I system:

e.      Dermatological system:

f.      Musculoskeletal system:

Others (specific):
